# Supplementary material for: IQSEC2 Deficiency Results in Abnormal Social Behaviors Relevant to Autism by Affecting Functions of Neural Circuits in the Medial Prefrontal Cortex
Source: Cells. 2021 Oct 12;10(10):2724. doi: 10.3390/cells10102724 (PMC8534507; doi:10.3390/cells10102724)
Supplement: Supplementary file 1 [file cells-10-02724-s001.zip › Supplementary Figure Legends.docx]

**SUPPLEMENTARY FIGURE LEGENDS**

**SUPPLE FIGURE 1: Generation of IQSEC2 KO mice using CRISPR/Cas9 genome editing.**

A. Genomic organization of IQSEC2 gene. Exon 3 to exon 13 are shared exons between isoform 1 and 2. sgRNA is designed within exon 3 that will target both isoforms. 17 bp are deleted in exon 3 as a result of NHEJ in one mouse. B. Genomic sequences around sgRNA in wild-type (left) and mutant mouse (right). C. Amino acid sequence near the mutated region and the domain structure of wild-type (top) and KO (bottom). Stop codon is introduced after Serine at position 254 in IQSEC2 KO mice that will produce truncated IQSEC protein after N-terminal CC region. D. Representative immunoblots for IQSEC2 (top) and β-Actin (bottom) antibodies using total brain homogenates from wild-type and IQSEC2 KO mice. E. Body weight (gm) of wild-type and IQSEC2 KO mice (WT n = 11, KO n = 11). Representative photo image of P33 IQSEC2 KO and wild-type (WT) mice showing IQSEC2 KO is smaller than wild-type mice.

Data in E and F are means ± SEM. Statistical analyses were performed by Student’s t test (**p < 0.01). Data values used in graphs are shown in Supplementary Table 2.

**SUPPLE FIGURE 2: AMPAR, NMDAR, and GABAR-mediated synaptic transmissions are decreased in pyramidal neurons in layer 5 of the mPFC in P35 IQSEC2 KO mice.**

A. Sample traces and summary graphs for mEPSCs in pyramidal neurons in layer 5 of the mPFC in wild-type (WT) and IQSEC2 KO (KO) mice. The frequency, but not amplitude, of mEPSCs is decreased in IQSEC2 KO mice. B. Sample traces and summary graphs for mIPSCs in pyramidal neurons in layer 5 of the mPFC in wild-type (WT) and IQSEC2 KO (KO) mice. The frequency, but not amplitude, of mIPSCs is decreased in IQSC2 KO mice. C. Sample traces and summary graph for the paired-pulse ratio of evoked EPSC in wild-type (WT) and IQSEC2 KO (KO) mice. The paired-pulse ratio is increased in IQSEC2 KO mice in 30, 50, and 100 ms stimulation intervals, suggesting that the release probability is decreased in the IQSEC2 KO neurons. D. Sample traces and summary graph for the paired-pulse ratio of evoked IPSC in wild-type (WT) and IQSEC2 KO (KO) mice. The paired-pulse ratio is unchanged in IQSEC2 KO mice. E. Sample traces and summary graph for evoked AMPA EPSC in wild-type (WT) and IQSEC2 KO (KO) mice. The amplitude of evoked AMPA EPSC is decreased in IQSEC2 KO mice. F. Sample traces and summary graph for evoked NMDA EPSC in wild-type (WT) and IQSEC2 KO (KO) mice. The amplitude of evoked NMDA EPSC is decreased in IQSEC2 KO mice. G. Sample traces and summary graph for evoked GABA IPSC in wild-type (WT) and IQSEC2 KO (KO) mice. The amplitude of evoked GABA IPSC is decreased in IQSEC2 KO mice. H. Sample traces and summary graph for the NMDA/AMPA ratio in wild-type (WT) and IQSEC2 KO (KO) mice. The NMDA/AMPA ratio is increased in IQSEC2 KO mice.

Data are means ± SEM (numbers of neurons/independent animals examined are shown in graphs). Statistical analyses were performed by Student’s t test (*p < 0.05; **p < 0.01; ***p < 0.001). Data values used in graphs are shown in Supplementary Table 2.

**SUPPLE FIGURE 3: Overexpression of IQSEC2 isoform 1 in the mPFC of wild-type mice does not affect behaviors.**

A. Representative immunofluorescent images of IQSEC2-AAV infected brain regions. Scale bar = 0.1 mm. B. Summary graphs for examining anxiety level in the elevated plus maze in control-AAV (Control) and IQSEC2-AAV (OE) infected mice. C. Summary graph for interaction with a juvenile mouse in control-AAV (Control) and IQSEC2-AAV (OE) infected mice. D. Heatmap images and summary graphs for social preference test in control-AAV (Control) and IQSEC2-AAV (OE) infected mice. E. Heatmap images and summary graphs for social novelty preference test in control-AAV (Control) and IQSEC2-AAV (OE) infected mice. F. Heatmap images and summary graph for novel object preference test in control-AAV (Control) and IQSEC2-AAV (OE) infected mice.

Data are means ± SEM (numbers of animals examined are shown in graphs). Statistical analyses were performed by Student’s t test (**p < 0.01; ***p < 0.001). Data values used in graphs are shown in Supplementary Table 2.

**SUPPLE FIGURE 4: Evaluation of the knockdown efficiency of IQSEC2 shRNA**

Summary graph for the knockdown efficiency for IQSEC2 shRNA examined by qPCR. IQSEC2 expression level is reduced to 17.04% in IQSEC2 shRNA viral infected neurons (KD) compared to the control infected neurons (Control). Control: n = 3, KD: n = 3.
