# Supplementary material for: IQSEC2 Deficiency Results in Abnormal Social Behaviors Relevant to Autism by Affecting Functions of Neural Circuits in the Medial Prefrontal Cortex
Source: Cells. 2021 Oct 12;10(10):2724. doi: 10.3390/cells10102724 (PMC8534507; doi:10.3390/cells10102724)
Supplement: Supplementary file 1 [file cells-10-02724-s001.zip › Table S1.DOCX]

Table S1: Oligo DNA sequences used in this study.

| Purpose | Code | 5’-3’ Sequence |
| --- | --- | --- |
| Genotyping Primer pair | KT20395 | TCCTGGCTCATTTTATCTCCTCC |
|  | KT20396 | GGGTTGGCTCCCAGGACTATC |
| Transgenic mouse Screening PCR Primer pair | AM-18001-IQSEC2 fwd | GAACCGTGTAGGCAGTGAAGA |
|  | AM-18002-IQSEC2 rev | ACTGTCCCTCCCTGAATACCA |
| WT / KO Sequencing Primer | AM-18002-IQSEC2 rev | ACTGTCCCTCCCTGAATACCA |
| Primer pair A | KT19359 | CGACGCGTCGAACGCTGACGTCATCAAC |
|  | KT19360 | CGACGCGTTTAATTAAGGCGCGCCCGGTGTTTCGTCCTTTCCACAAG |
| Primer pair B | Flag_IQSEC2_F | ACCGGTGCCACCATGGACTACAAAGACGATGACGA |
|  | IQSEC2_R | TCTTTTATTGAATTCTCAGACCACAGTGCTGA |
| Primer Pair C | IQSEC2_shR_F | GCAATTTACCGAGATAAGGAGCGAGAAGCTTCC |
|  | IQSEC2_shR_R | ATCTCGGTAAATTGCTTCCCGGTTCTGGTAAGC |
| shIQSEC2 oligo DNAs | KT19351 | TCGACCCGGAAGCTATCTATCGGGATAATTCAAGAGATTATCCCGATAGATAGCTTCCTTTTTTGGAAAT |
|  | KT19352 | CTAGATTTCCAAAAAAGGAAGCTATCTATCGGGATAATCTCTTGAATTATCCCGATAGATAGCTTCCGGG |
| Primers for sequencing IQSEC2 cDNA | KT19374 | CGGAGGTCACAGCACCAGTAC |
|  | KT19375 | CCTCCACACTGACTGTTCTG |
|  | IQSEC2_seq2R | CTTCCTCACGGCTACCATCTTC |
|  | IQSEC2_seq3R | CCATAGAGGAGAAGTCCATCTC |
|  | IQSEC2_seq4F | GCCTTCAACAATGATGTGG |
|  | IQSEC2_seq4R | CATGGGCATGGAAGTGTCGATG |
|  | IQSEC2_seq5F | TCGCTCCCTCACCGTCGACTGGTTTG |
|  | IQSEC2_3290F | TGCGGTACCTGGAGGGGAACGAAAAGTTCTCAT |
|  | IQSEC2_4200F | CCTCAGTTTGCTCCACATGGCCGCCACCCC |
|  | IQSEC2_4380R | GTAGGATCCCCCTGGTGGCCGGGACCCAGGGCC |
|  | EFS1_F | GATGTCGTGTACTGGCTCCGCC |
|  | EFS_CTL | GTCAGTGGGCAGAGCGCACATCGC |
| qPCR primers for AAV titration  (YS002) | KT18345 | AAGGGCGAGGAGCTGTTCAC |
|  | KT99100 | TGCAGATGAACTTCAGGGTC |
| qPCR primers for AAV titration  (YS007) | KT18345 | AAGGGCGAGGAGCTGTTCAC |
|  | KT99100 | TGCAGATGAACTTCAGGGTC |
| qPCR primers for AAV titration  (AM004) | KT19374 | CGGAGGTCACAGCACCAGTAC |
|  | KT19375 | CCTCCACACTGACTGTTCTG |
| qPCR primers for AAV titration  (AM006) | EFS1_F | GATGTCGTGTACTGGCTCCGCC |
|  | EFS1_R | GTGGCACCGGTCCTGTGTTCTG |
| Knockdown efficiency  IQSEC2 | KT19374 | CGGAGGTCACAGCACCAGTAC |
|  | KT19375 | CCTCCACACTGACTGTTCTG |
| Knockdown efficiency  GAPDH | GAPDHfwd | CATGGCCTTCCGTGTTCCTA |
|  | GAPDHrev | CCTGCTTCACCACCTTCTTGA |
