# Supplementary material for: IQSEC2 Deficiency Results in Abnormal Social Behaviors Relevant to Autism by Affecting Functions of Neural Circuits in the Medial Prefrontal Cortex
Source: Cells. 2021 Oct 12;10(10):2724. doi: 10.3390/cells10102724 (PMC8534507; doi:10.3390/cells10102724)
Supplement: Supplementary file 1 [file cells-10-02724-s001.zip › Table S2.DOCX]

Table S2: Results of Data Analysis (1).

| Figure | Parameter | Sample | Mean ± SEM | Statistical Tests |
| --- | --- | --- | --- | --- |
| S1E | Body Weight at P3 | WT | 2.21090 ± 0.1118 gm | Student’s t-test  P= 0.006075 |
|  |  | KO | 1.83818 ± 0.0475 gm |  |
|  | Body Weight at P5 | WT | 2.9772 ± 0.0917 gm | Student’s t-test  P= 0.00524 |
|  |  | KO | 2.77 ± 0.160 gm |  |
|  | Body Weight at P7 | WT | 3.57909 ± 0.1307 gm | Student’s t-test  P= 0.017894 |
|  |  | KO | 3.2627 ± 0.1827 gm |  |
|  | Body Weight at 9 | WT | 4.24818 ± 0.2238 gm | Student’s t-test  P= 0.033128 |
|  |  | KO | 3.9572 ± 0.2816 gm |  |
|  | Body Weight at P11 | WT | 5.0172 ± 0.3133 gm | Student’s t-test  P= 0.018911 |
|  |  | KO | 4.3427 ± 0.259 gm |  |
|  | Body Weight at P13 | WT | 5.3754 ± 0.35 gm | Student’s t-test  P= 0.012243 |
|  |  | KO | 4.51 ± 0.255217 gm |  |
|  | Body Weight at P14 | WT | 5.645 ± 0.366612 gm | Student’s t-test  P= 0.027661 |
|  |  | KO | 4.7282 ± 0.23309 gm |  |
| 1A | Travel Distance (0-10 min) | WT | 4905.26 ± 268.8 cm | Student’s t-test  P= 0.114143 |
|  |  | KO | 6045.89 ± 652.2 cm |  |
|  | Travel Distance (0-10 min) | WT | 3656.89 ± 168.86 cm | Student’s t-test  P= 0.002237 |
|  |  | KO | 5529.79 ± 537.55 cm |  |
|  | Travel Distance (0-10 min) | WT | 3246.58 ± 155.74 cm | Student’s t-test  P= 0.00403 |
|  |  | KO | 4883.66 ± 506.32 cm |  |
|  | Time Spent in Centre | WT | 106 ± 14.004 sec | Student’s t-test  P= 0.082805 |
|  |  | KO | 66.3846 ± 15.98 sec |  |
|  | Vertical Activity | WT | 220.67 ± 15.73 | Student’s t-test  P= 0.115539 |
|  |  | KO | 282.77 ± 35.062 |  |
|  | Grooming (0-10 min) | WT | 16.27 ± 2.005 sec | Student’s t-test  P= 0.065985 |
|  |  | KO | 37.769 ± 11.827 sec |  |
|  | Grooming (10-20 min) | WT | 48.4 ± 7.622073 sec | Student’s t-test  P= 0.092222 |
|  |  | KO | 92.38 ± 25.51333 sec |  |
|  | Grooming (20-30 min) | WT | 42.6 ± 5.785653 sec | Student’s t-test  P= 0.016615 |
|  |  | KO | 121.23 ± 31.0806 sec |  |
| 1B | Open arm Time | WT | 45.402 ± 5.19 % | Student’s t-test  P= 0.938176 |
|  |  | KO | 44.66642 ± 7.65 % |  |
|  | Closed Arm Time | WT | 54.59823 ± 5.19 % | Student’s t-test  P= 0.938176 |
|  |  | KO | 55.33358 ± 7.65 % |  |
| 1C | Sociability  Time Spent by WT | S1 | 241.6 ± 23.35043 sec | Student’s t-test  P= 4.52838E-07 |
|  |  | E | 70.73333 ± 9.75 sec |  |
|  | Sociability  Time Spent by KO | S1 | 61 ± 8.437027 sec | Student’s t-test  P= 0.804142 |
|  |  | E | 64.46154 ± 10.23 sec |  |
|  | Sociability  (S1-E) | WT | 170.8667 ± 31.35 sec | Student’s t-test  P= 5.0468E-05 |
|  |  | KO | -3.46154 ± 8.835 sec |  |
| 1D | Social Novelty  Time Spent by WT | S1 | 97.53333 ± 12.66 sec | Student’s t-test  P= 0.001869 |
|  |  | S2 | 187.4 ± 21.88 sec |  |
|  | Social Novelty  Time Spent by KO | S1 | 44.08333 ± 7.096 sec | Student’s t-test  P= 0.451541 |
|  |  | S2 | 52.91667 ± 7.88 sec |  |
|  | Social Novelty  (S2-S1) | WT | 89.86667 ± 27.24 sec | Student’s t-test  P= 0.019759 |
|  |  | KO | 8.833333 ± 7.791 sec |  |
| 1E | Interaction time with Juvenile mouse | WT | 219.7143 ± 20.34 sec | Student’s t-test  P= 0.035233 |
|  |  | KO | 154.25 ± 19.065 sec |  |
| 1F | Novel Object Recognition  Time Spent by WT | A3 | 8 ± 1.28 sec | Student’s t-test  P= 0.006859 |
|  |  | B | 20.06667 ± 3.78 sec |  |
|  | Novel Object Recognition  Time Spent by KO | A3 | 17 ± 3.458973 sec | Student’s t-test  P= 9.96796E-07 |
|  |  | B | 68.15385 ± 6.716 sec |  |
|  | Novel Object Recognition  (B-A3) | WT | 12.06667 ± 3.569583 | Student’s t-test  P= 3.06E-05 |
|  |  | KO | 51.15385 ± 6.899829 |  |
| 3A | Frequency of mEPSC | WT | 1.968 ± 0.247 Hz | Student’s t-test  P= 3.53968E-05 |
|  |  | KO | 0.41 ± 0.102 Hz |  |
|  | Amplitude of mEPSC | WT | 8.580 ± 0.269 pA | Student’s t-test  P= 0.78256135 |
|  |  | KO | 8.455 ± 0.333 pA |  |
| 3B | Frequency of mIPSC | WT | 1.992 ± 0.345 Hz | Student’s t-test  P= 0.011645 |
|  |  | KO | 0.87 ± 0.219 Hz |  |
|  | Amplitude of mIPSC | WT | 12.43 ± 0.508 pA | Student’s t-test  P= 0.097648 |
|  |  | KO | 10.819 ± 0.635 pA |  |
| 3C | excitatory PPR  30 ms Pulse interval | WT | 1.128381 ± 0.048509 | Student’s t-test  P= 0.004345 |
|  |  | KO | 1.454298 ± 0.083732 |  |
|  | excitatory PPR  50 ms Pulse interval | WT | 1.036624 ± 0.051053 | Student’s t-test  P= 0.004012 |
|  |  | KO | 1.353339 ± 0.078207 |  |
|  | excitatory PPR  100 ms Pulse interval | WT | 1.001336 ± 0.058995 | Student’s t-test  P= 0.00179 |
|  |  | KO | 1.299716 ± 0.055681 |  |
| 3D | Inhibitory PPR  30 ms | WT | 0.550419 ± 0.043109 | Student’s t-test  P= 0.952891 |
|  |  | KO | 0.554173 ± 0.039978 |  |
|  | Inhibitory PPR  50 ms | WT | 0.647972 ± 0.053912 | Student’s t-test  P= 0.736399 |
|  |  | KO | 0.623637 ± 0.034149 |  |
|  | Inhibitory PPR  100 ms | WT | 0.732939 ± 0.035503 | Student’s t-test  P= 0.633872 |
|  |  | KO | 0.755109 ± 0.019468 |  |
|  | Inhibitory PPR  200 ms | WT | 0.834239 ± 0.021432 | Student’s t-test  P= 0.058093 |
|  |  | KO | 0.761917 ± 0.028831 |  |
|  | Inhibitory PPR  500 ms | WT | 0.782771 ± 0.017976 | Student’s t-test  P= 0.692679 |
| 3E | AMPA EPSC Amplitude | WT | 818.1 ± 150.6658 pA | Student’s t-test  P= 0.03819 |
|  |  | KO | 427.4 ± 68.97118 pA |  |
| 3F | NMDA EPSC Amplitude | WT | 321.608 ± 41.859 pA | Student’s t-test  P= 0.025947 |
|  |  | KO | 185.2667 ± 35.15 pA |  |
| 3G | GABA EPSC Amplitude | WT | 283.33 ± 39.898 pA | Student’s t-test  P= 0.004457 |
|  |  | KO | 110.806 ± 30.759 pA |  |
| 3H | NMDA/AMPA ratio | WT | 0.872879 ± 0.124442 | Student’s t-test  P= 0.043076 |
|  |  | KO | 1.354894 ± 0.175267 |  |
| S2A | Frequency of mEPSC | WT | 7.5402 ± 0.7058 Hz | Student’s t-test  P= 2.12E-06 |
|  |  | KO | 2.5773 ± 0.1858 Hz |  |
|  | Amplitude of mEPSC | WT | 6.889835 ± 0.185 pA | Student’s t-test  P= 0.702224 |
|  |  | KO | 7.020621 ± 0.243 pA |  |
| S2B | Frequency of mIPSC | WT | 4.51556 ± 0.6095 Hz | Student’s t-test  P= 0.006756 |
|  |  | KO | 2.1952 ± 0.2859 Hz |  |
|  | Amplitude of mIPSC | WT | 13.58164 ± 0.6427pA | Student’s t-test  P= 0.074399 |
|  |  | KO | 15.81571 ± 0.878 pA |  |
| S2C | excitatory PPR  30 ms Pulse interval | WT | 1.002326 ± 0.059506 | Student’s t-test  P= 0.021312 |
|  |  | KO | 1.324703 ± 0.114307 |  |
|  | excitatory PPR  50 ms Pulse interval | WT | 1.065444 ± 0.046458 | Student’s t-test  P = 0.019606 |
|  |  | KO | 1.357326 ± 0.106437 |  |
|  | excitatory PPR  100 ms Pulse interval | WT | 0.990744 ± 0.032317 | Student’s t-test  P = 0.04303 |
|  |  | KO | 1.196652 ± 0.091669 |  |
| S2D | Inhibitory PPR  30 ms | WT | 0.356829 ± 0.032816 | Student’s t-test  P = 0.6648 |
|  |  | KO | 0.33042 ± 0.048416 |  |
|  | Inhibitory PPR  50 ms | WT | 0.48696 ± 0.03774 | Student’s t-test  P = 0.248958 |
|  |  | KO | 0.410788 ± 0.049616 |  |
|  | Inhibitory PPR  100 ms | WT | 0.630365 ± 0.043853 | Student’s t-test  P = 0.188805 |
|  |  | KO | 0.53423 ± 0.052586 |  |
|  | Inhibitory PPR  200 ms | WT | 0.675615 ± 0.034083 | Student’s t-test  P = 0.231638 |
|  |  | KO | 0.611831 ± 0.036371 |  |
|  | Inhibitory PPR  500 ms | WT | 0.764834 ± 0.015304 | Student’s t-test  P = 0.152275 |
|  |  | KO | 0.700425 ± 0.040139 |  |
| S2E | AMPA EPSC Amplitude | WT | 568.083 ± 112.93 pA | Student’s t-test  P = 0.044453 |
|  |  | KO | 279.1091 ± 54.98 pA |  |
| S2F | NMDA EPSC Amplitude | WT | 266.889 ± 42.877 pA | Student’s t-test  P = 0.01094 |
|  |  | KO | 131.005 ± 20.052 pA |  |
| S2G | GABA EPSC Amplitude | WT | 1136.74 ± 96.727 pA | Student’s t-test  P = 0.000153 |
|  |  | KO | 540.617 ± 79.166 pA |  |
| S2H | NMDA/AMPA ratio | WT | 0.660202 ± 0.106124 | Student’s t-test  P = 0.012108 |
|  |  | KO | 1.458094 ± 0.248308 |  |
| 4C | Frequency of mEPSC | Control | 2.71797 ± 0.4082 Hz | Student’s t-test  P= 0.002762 |
|  |  | KD | 1.14677 ± 0.2113 Hz |  |
|  | Amplitude of mEPSC | Control | 9.0657 ± 0.2415 pA | Student’s t-test  P= 0.002471 |
|  |  | KD | 8.0010 ± 0.1836 pA |  |
| 4D | Frequency of mIPSC | Control | 2.347345 ± 0.30 Hz | Student’s t-test  P= 0.009824 |
|  |  | KD | 1.34794 ± 0.1789 Hz |  |
|  | Amplitude of mIPSC | Control | 13.317 ± 0.8067 pA | Student’s t-test  P= 0.837927 |
|  |  | KD | 13.078 ± 0.7571 pA |  |
| 4E | excitatory PPR | Control | 1.179957 ± 0.049427 | Student’s t-test  P= 0.032902 |
| 4F | AMPA EPSC Amplitude | Control | 1499 ± 195.7841 pA | Student’s t-test  P= 0.017951 |
|  |  | KD | 834.5 ± 142.4072 pA |  |
| 4G | NMDA EPSC Amplitude | Control | 229.67 ± 40.493 pA | Student’s t-test  P= 0.013305 |
|  |  | KD | 107.57 ± 19.789 pA |  |
|  | NMDA EPSC Amplitude | Control | 495.77 ± 79.425 pA | Student’s t-test  P= 0.0305 |
|  |  | KD | 278.67 ± 47.588 pA |  |
|  | NMDA EPSC Amplitude | Control | 633.18 ± 99.989 pA | Student’s t-test  P= 0.014423 |
|  |  | KD | 346.3371 ± 40.15 pA |  |
| 4H | GABA IPSC Amplitude  0.2 mA | Control | 726.725 ± 94.581 pA | Student’s t-test  P= 0.000222 |
|  |  | KD | 187.446 ± 53.156 pA |  |
|  | GABA IPSC Amplitude  0.4 mA | Control | 1264.05 ± 170.42 pA | Student’s t-test  P= 0.004993 |
|  |  | KD | 536.424 ± 127.42 pA |  |
|  | GABA IPSC Amplitude  1 mA | Control | 1547.72 ± 189.20 pA | Student’s t-test  P= 0.035973 |
|  |  | KD | 925.43 ± 178.91 pA |  |
| 4I | NMDA/AMPA Ratio | Control | 1.229919 ± 0.132875 | Student’s t-test  P= 0.00165 |
| 5B | Frequency of mEPSC | WT Control | 4.7545 ± 0.3834 Hz | one-way ANOVA Bonferroni posthoc  P= 6.23E-07 vs KO Control |
|  |  | KO Control | 1.7172 ± 0.2157 Hz | one-way ANOVA Bonferroni posthoc  P= 7.9E-7 vs KO Rescue |
|  |  | KO Rescue | 4.5105 ± 0.260 Hz | one-way ANOVA Bonferroni posthoc  P>0.9999 vs WT Control |
|  | Amplitude of mEPSCs | WT Control | 7.9546 ± 0.3064 pA | one-way ANOVA Bonferroni posthoc  P= 0.237 vs KO Control |
|  |  | KO Control | 9.407 ± 0.7317 pA | one-way ANOVA Bonferroni posthoc  P= 0.69 vs KO Rescue |
|  |  | KO Rescue | 8.4979 ± 0.3999 pA | one-way ANOVA Bonferroni posthoc  P>0.9999 vs WT Control |
| 5C | Frequency of mIPSC | WT Control | 2.8037 ± 0.3237 Hz | one-way ANOVA Bonferroni posthoc  P= 0.0007 vs KO Control |
|  |  | KO Control | 1.3395 ± 0.1598 Hz | one-way ANOVA Bonferroni posthoc  P= 0.012 vs KO Rescue |
|  |  | KO Rescue | 2.383 ± 0.182 Hz | one-way ANOVA Bonferroni posthoc  P=0.662 vs WT Control |
|  | Amplitude of mIPSCs | WT Control | 13.806 ± 0.84354 pA | one-way ANOVA Bonferroni posthoc  P>0.9999 vs KO Control |
|  |  | KO Control | 15.485 ± 1.3396 pA | one-way ANOVA Bonferroni posthoc  P>0.9999 vs KO Rescue |
|  |  | KO Rescue | 16.358 ± 1.11355 pA | one-way ANOVA Bonferroni posthoc  P=0.39 vs WT Control |
| 5D | excitatory PPR | WT Control | 0.966515 ± 0.056094 | one-way ANOVA Bonferroni posthoc  P= 0.0017 vs KO Control |
|  |  | KO Control | 1.456312 ± 0.104384 | one-way ANOVA Bonferroni posthoc  P= 0.033 vs KO Rescue |
|  |  | KO Rescue | 1.106904 ± 0.095213 | one-way ANOVA Bonferroni posthoc  P= 0.825 vs WT Control |
| 5E | AMPA EPSC Amplitude | WT Control | 384.69 ± 49.563 pA | one-way ANOVA Bonferroni posthoc  P= 0.014 vs KO Control |
|  |  | KO Control | 174.83 ± 22.8459 pA | one-way ANOVA Bonferroni posthoc  P= 0.0108 vs KO Rescue |
|  |  | KO Rescue | 420.9 ± 78.95156 pA | one-way ANOVA Bonferroni posthoc  P>0.9999 vs WT Control |
| 5F | GABA IPSC Amplitude | WT Control | 494.7 ± 101.5775 pA | one-way ANOVA Bonferroni posthoc  P= 0.018 vs KO Control |
|  |  | KO Control | 164.684 ± 24.206 pA | one-way ANOVA Bonferroni posthoc  P= 0.037 vs KO Rescue |
|  |  | KO Rescue | 478.35 ± 52.9476 pA | one-way ANOVA Bonferroni posthoc  P>0.9999 vs WT Control |
| 5G | NMDA/AMPA ratio | WT Control | 0.876458 ± 0.08845 | one-way ANOVA Bonferroni posthoc  P= 0.017 vs KO Control |
|  |  | KO Control | 1.662366 ± 0.267252 | one-way ANOVA Bonferroni posthoc  P= 0.018 vs KO Rescue |
|  |  | KO Rescue | 0.826385 ± 0.178438 | one-way ANOVA Bonferroni posthoc  P= 0.89 vs WT Control |
| 6A | Interaction time with Juvenile mouse | WT Control | 224.46 ± 18.7148 sec | one-way ANOVA Tukey’s posthoc  P= 0.0109 vs KO Control |
|  |  | KO Control | 127.83 ± 21.7395 sec | one-way ANOVA Tukey’s posthoc  P= 0.044 vs KO Rescue |
|  |  | KO Rescue | 209.7 ± 24.741 sec | one-way ANOVA Tukey’s posthoc  P= 0.89 vs WT Control |
| 6B | Sociability  Time Spent by WT Control | S1 | 248.846 ± 24.896 sec | Student’s t-test  P = 3.36876E-07 |
|  |  | E | 60.308 ± 7.563 sec |  |
|  | Sociability  Time Spent by KO Control | S1 | 78.917 ± 20.407 sec | Student’s t-test  P = 0.255299 |
|  |  | E | 125.167 ± 31.95 sec |  |
|  | Sociability  Time Spent by KO Rescue | S1 | 130.25 ± 23.18 sec | Student’s t-test  P = 0.006582 |
|  |  | E | 49.4167 ± 11.304 sec |  |
|  | Sociability  (S1-E) | WT Control | 188.54 ± 26.652 sec | one-way ANOVA Tukey’s posthoc  P= 0 .001005 vs KO Control |
|  |  | KO Control | -46.25 ± 43.346 sec | one-way ANOVA Tukey’s posthoc  P= 0.04 vs KO Rescue |
|  |  | KO Rescue | 80.833 ± 28.956 sec | one-way ANOVA Tukey’s posthoc  P= 0.87 vs WT Control |
| 6C | Social Novelty  Time Spent by WT Control | S1 | 102.077 ± 12.489 sec | Student’s t-test  P = 0.000114 |
|  |  | E | 185 ± 11.98 sec |  |
|  | Social Novelty  Time Spent by KO Control | S1 | 79.33 ± 17.939 sec | Student’s t-test  P = 0.29804 |
|  |  | E | 56.9167 ± 9.1465 sec |  |
|  | Social Novelty  Time Spent by KO Rescue | S1 | 44.67 ± 10.61467 sec | Student’s t-test  P = 0.087018 |
|  |  | E | 39.75 ± 12.35 sec |  |
|  | Social Novelty  (S2-S1) | WT Control | 82.923 ± 22.558 sec | one-way ANOVA Tukey’s posthoc  P= 0 .001005 vs KO Control |
|  |  | KO Control | -22.42 ± 12.557 sec | one-way ANOVA Tukey’s posthoc  P= 0.049 vs KO Rescue |
|  |  | KO Rescue | 39.75 ± 12.34578 sec | one-way ANOVA Tukey’s posthoc  P= 0.2 vs WT Control |
| S3B | Open arm Time | WT Control | 55.5755 ± 3.753 % | Student’s t-test  P= 0.093411 |
|  |  | OE | 41.60328 ± 6.891 % |  |
|  | Open Arm Entries | WT Control | 12.85714 ± 1.004 | Student’s t-test  P= 0.466436 |
|  |  | OE | 11.54545 ± 1.367 |  |
|  | Closed Arm Time | WT Control | 44.4245 ± 3.753 % | Student’s t-test  P= 0.093411 |
|  |  | OE | 58.39672 ± 6.89 % |  |
|  | Closed Arm Entries | WT Control | 10.07143 ± 1.002366 | Student’s t-test  P= 0.891379 |
|  |  | OE | 10.27273 ± 0.888401 |  |
| S3C | Interaction time with Juvenile mouse | WT Control | 224.46 ± 18.715 sec | Student’s t-test  P= 0.565426 |
|  |  | OE | 239.36 ± 14.737 sec |  |
| S3D | Sociability  Time Spent by WT Control | S1 | 248.846 ± 24.89 sec | Student’s t-test  P= 3.36876E-07 |
|  |  | E | 60.308 ± 7.563 sec |  |
|  | Sociability  Time Spent by OE | S1 | 260.6364 ± 17.62 sec | Student’s t-test  P= 2.36246E-07 |
|  |  | E | 101.8182 ± 9.078 sec |  |
|  | Sociability  (S1-E) | WT | 188.5385 ± 26.65 sec | Student’s t-test  P= 0.443343 |
|  |  | KO | 158.82 ± 24.04 sec |  |
| S3E | Social Novelty  Time Spent by  WT Control | S1 | 102.08 ± 12.489 sec | Student’s t-test  P= 0.000114 |
|  |  | S2 | 185 ± 11.9798 sec |  |
|  | Social Novelty  Time Spent by OE | S1 | 111.36 ± 15.4595 sec | Student’s t-test  P= 0.001194 |
|  |  | S2 | 187.36 ± 11.393 sec |  |
|  | Social Novelty  (S2-S1) | WT Control | 82.923 ± 22.558 sec | Student’s t-test  P= 0.830679 |
|  |  | OE | 76 ± 19.954 sec |  |
| S3F | Novel Object Recognition  Time Spent by  WT Control | A3 | 7.5833 ± 3.16328 sec | Student’s t-test  P= 0.66037 |
|  |  | B | 9.33 ± 2.035563 sec |  |
|  | Novel Object Recognition  Time Spent by OE | A3 | 7 ± 2.222611 sec | Student’s t-test  P= 0.924632 |
|  |  | B | 7.3 ± 1.964943 sec |  |
| S4 | Knockdown Efficiency | Control | 100 ± 0 % | - |
|  |  | KD | 17.4 ± 1.77 % |  |
