# Supplementary material for: IQSEC2 Deficiency Results in Abnormal Social Behaviors Relevant to Autism by Affecting Functions of Neural Circuits in the Medial Prefrontal Cortex
Source: Cells. 2021 Oct 12;10(10):2724. doi: 10.3390/cells10102724 (PMC8534507; doi:10.3390/cells10102724)
Supplement: Supplementary file 1 [file cells-10-02724-s001.zip › Table S3.docx]

Table S3: Statistical Data for cFos mapping.

| Stained C-FOS cells /mm^2 in Infralimbic Cortex (IL) | | | | | | | | |
| --- | --- | --- | --- | --- | --- | --- | --- | --- |
|  | Mean ± SEM | Statistics | WT(B)  vs KO(B) | KO(B)  vs KO | KO(B)  vs WT | WT(B)  vs KO | WT(B)  vs WT | WT  vs KO |
| WT | 36.98659 ± 5.37797 | One- way ANOVA, Tukey’s posthoc,  F_3,8_= 13.02, P= 0.0019 | P= 0 .049 | P= 0 .224 | P= 0 .189 | P= 0 .003 | P= 0 .003 | P= 0 .89 |
| KO | 38.95044 ± 3.7606 |  |  |  |  |  |  |  |
| WT (B) | 119.9968 ± 14.80 |  |  |  |  |  |  |  |
| KO (B) | 71.23683 ± 6.7762 |  |  |  |  |  |  |  |
| Stained C-FOS cells /mm^2 in Prilimbic Cortex (PrL) | | | | | | | | |
|  | Mean ± SEM | Statistics | WT(B)  vs KO(B) | KO(B)  vs KO | KO(B)  vs WT | WT(B)  vs KO | WT(B)  vs WT | WT  vs KO |
| WT | 30.21335 ± 2.0228 | One- way ANOVA, Tukey’s posthoc,  F_3,8_= 10.37, P= 0.0039 | P= 0 .023 | P= 0 .83 | P= 0 .61 | P= 0 .008 | P= 0 .005 | P= 0 .89 |
| KO | 38.1025 ± 1.096677 |  |  |  |  |  |  |  |
| WT (B) | 125.0228 ± 21.556 |  |  |  |  |  |  |  |
| KO (B) | 53.6039 ± 3.460706 |  |  |  |  |  |  |  |
| Stained C-FOS cells /mm^2 in cingulate cortex, area 1 (CG1) | | | | | | | | |
|  | Mean ± SEM | Statistics | WT(B)  vs KO(B) | KO(B)  vs KO | KO(B)  vs WT | WT(B)  vs KO | WT(B)  vs WT | WT  vs KO |
| WT | 46.1451 ± 2.347767 | One- way ANOVA, Tukey’s posthoc,  F_3,8_= 25.55, P= 0.0002 | P= 0 .003 | P= 0 .26 | P= 0 .16 | P= 0 .001 | P= 0 .001 | P= 0 .89 |
| KO | 50.45809 ± 2.0715 |  |  |  |  |  |  |  |
| WT (B) | 139.77 ± 13.2787 |  |  |  |  |  |  |  |
| KO (B) | 74.72649 ± 2.9498 |  |  |  |  |  |  |  |
| Stained C-FOS cells /mm^2 in cingulate cortex, area 2 (CG2) | | | | | | | | |
|  | Mean ± SEM | Statistics | WT(B)  vs KO(B) | KO(B)  vs KO | KO(B)  vs WT | WT(B)  vs KO | WT(B)  vs WT | WT  vs KO |
| WT | 45.757 ± 2.67741 | One- way ANOVA, Tukey’s posthoc,  F_3,8_= 24.54, P= 0.0002 | P= 0 .008 | P= 0 .102 | P= 0 .071 | P= 0 .001 | P= 0 .001 | P= 0 .89 |
| KO | 49.9584 ± 5.8103 |  |  |  |  |  |  |  |
| WT (B) | 170.147 ± 16.14909 |  |  |  |  |  |  |  |
| KO (B) | 94.377 ± 7.7852 |  |  |  |  |  |  |  |
| Stained C-FOS cells /mm^2 in lateral septal nucleus (LS) | | | | | | | | |
|  | Mean ± SEM | Statistics | WT(B)  vs KO(B) | KO(B)  vs KO | KO(B)  vs WT | WT(B)  vs KO | WT(B)  vs WT | WT  vs KO |
| WT | 51.587 ± 5.1936 | One- way ANOVA, Tukey’s posthoc,  F_3,8_= 6.4, P= 0.0157 | P= 0 .89 | P= 0 .043 | P= 0 .041 | P= 0 .083 | P= 0 .077 | P= 0 .89 |
| KO | 52.64796 ± 8.74518 |  |  |  |  |  |  |  |
| WT (B) | 114.88 ± 22.06675 |  |  |  |  |  |  |  |
| KO (B) | 124.67 ± 6.810103 |  |  |  |  |  |  |  |
| Stained C-FOS cells /mm^2 in insular Cortex (Ins) | | | | | | | | |
|  | Mean ± SEM | Statistics | WT(B)  vs KO(B) | KO(B)  vs KO | KO(B)  vs WT | WT(B)  vs KO | WT(B)  vs WT | WT  vs KO |
| WT | 24.71978 ± 2.0195 | One- way ANOVA, Tukey’s posthoc,  F_3,8_= 4.553, P= 0.038 | P= 0 .44 | P= 0 .121 | P= 0 .031 | P= 0 .74 | P= 0 .28 | P= 0 .76 |
| KO | 43.152 ± 4.75747 |  |  |  |  |  |  |  |
| WT (B) | 62.2348 ± 9.42519 |  |  |  |  |  |  |  |
| KO (B) | 92.596 ± 19.408 |  |  |  |  |  |  |  |
| Stained C-FOS cells /mm^2 in claustrum (Cs) | | | | | | | | |
|  | Mean ± SEM | Statistics | WT(B)  vs KO(B) | KO(B)  vs KO | KO(B)  vs WT | WT(B)  vs KO | WT(B)  vs WT | WT  vs KO |
| WT | 76.0646 ± 8.48258 | One- way ANOVA, Tukey’s posthoc,  F_3,8_= 0.575,  P= 0.647 | P= 0 .89 | P= 0 .89 | P= 0 .62 | P= 0 .89 | P= 0 .89 | P= 0 .74 |
| KO | 109.9 ± 15.04251 |  |  |  |  |  |  |  |
| WT (B) | 99.01 ± 13.53374 |  |  |  |  |  |  |  |
| KO (B) | 117.68 ± 32.22573 |  |  |  |  |  |  |  |
| Stained C-FOS cells /mm^2 in bed nucleus of the stria terminalis, dorsal part (dBNST) | | | | | | | | |
|  | Mean ± SEM | Statistics | WT(B)  vs KO(B) | KO(B)  vs KO | KO(B)  vs WT | WT(B)  vs KO | WT(B)  vs WT | WT  vs KO |
| WT | 67.3627 ± 11.3279 | One- way ANOVA, Tukey’s posthoc,  F_3,8_= 4.31, P= 0.044 | P= 0 .77 | P= 0 .08 | P= 0 .07 | P= 0 .29 | P= 0 .24 | P= 0 .89 |
| KO | 70.383 ± 5.587116 |  |  |  |  |  |  |  |
| WT (B) | 115.3 ± 20.88755 |  |  |  |  |  |  |  |
| KO (B) | 137.083 ± 11.4365 |  |  |  |  |  |  |  |
| Stained C-FOS cells /mm^2 in bed nucleus of the stria terminalis, ventral part (vBNST) | | | | | | | | |
|  | Mean ± SEM | Statistics | WT(B)  vs KO(B) | KO(B)  vs KO | KO(B)  vs WT | WT(B)  vs KO | WT(B)  vs WT | WT  vs KO |
| WT | 97.526 ± 9.20657 | One- way ANOVA, Tukey’s posthoc,  F_3,8_= 1.815, P= 0.224 | P= 0 .82 | P= 0 .41 | P= 0 .21 | P= 0 .83 | P= 0 .56 | P= 0 .89 |
| KO | 112.965 ± 18.158 |  |  |  |  |  |  |  |
| WT (B) | 136.509 ± 25.1883 |  |  |  |  |  |  |  |
| KO (B) | 160.536 ± 8.25235 |  |  |  |  |  |  |  |
| Stained C-FOS cells /mm^2 in lateral amygdale (LA) | | | | | | | | |
|  | Mean ± SEM | Statistics | WT(B)  vs KO(B) | KO(B)  vs KO | KO(B)  vs WT | WT(B)  vs KO | WT(B)  vs WT | WT  vs KO |
| WT | 62.0131 ± 10.048 | One- way ANOVA, Tukey’s posthoc,  F_3,8_= 2.26, P= 0.158 | P= 0 .89 | P= 0 .25 | P= 0 .22 | P= 0 .50 | P= 0 .46 | P= 0 .89 |
| KO | 65.0479 ± 6.60192 |  |  |  |  |  |  |  |
| WT (B) | 109.98 ± 20.10647 |  |  |  |  |  |  |  |
| KO (B) | 128.416 ± 27.1939 |  |  |  |  |  |  |  |
| Stained C-FOS cells /mm^2 in central amygdale (CeA) | | | | | | | | |
|  | Mean ± SEM | Statistics | WT(B)  vs KO(B) | KO(B)  vs KO | KO(B)  vs WT | WT(B)  vs KO | WT(B)  vs WT | WT  vs KO |
| WT | 86.2814 ± 22.229 | One- way ANOVA, Tukey’s posthoc,  F_3,8_= 4.62, P= 0.037 | P= 0 .89 | P= 0 .083 | P= 0 .134 | P= 0 .095 | P= 0 .153 | P= 0 .89 |
| KO | 71.3801 ± 4.79583 |  |  |  |  |  |  |  |
| WT (B) | 191.207 ± 43.6847 |  |  |  |  |  |  |  |
| KO (B) | 195.317 ± 10.7459 |  |  |  |  |  |  |  |
| Stained C-FOS cells /mm^2 in posterior paraventricular thalamus (pPVT) | | | | | | | | |
|  | Mean ± SEM | Statistics | WT(B)  vs KO(B) | KO(B)  vs KO | KO(B)  vs WT | WT(B)  vs KO | WT(B)  vs WT | WT  vs KO |
| WT | 89.2433 ± 26.987 | One- way ANOVA, Tukey’s posthoc,  F_3,8_= 4.38, P= .042 | P= 0 .89 | P= 0 .119 | P= 0 .108 | P= 0 .14 | P= 0 .127 | P= 0 .89 |
| KO | 95.0445 ± 10.7954 |  |  |  |  |  |  |  |
| WT (B) | 298.2918 ± 54.21 |  |  |  |  |  |  |  |
| KO (B) | 307.7275 ± 72.488 |  |  |  |  |  |  |  |
| Stained C-FOS cells /mm^2 in lateral habenular nucleus (lHab) | | | | | | | | |
|  | Mean ± SEM | Statistics | WT(B)  vs KO(B) | KO(B)  vs KO | KO(B)  vs WT | WT(B)  vs KO | WT(B)  vs WT | WT  vs KO |
| WT | 75.376 ± 7.8618 | One- way ANOVA, Tukey’s posthoc,  F_3,8_= 2.60, P= 0.124 | P= 0 .41 | P= 0 .79 | P= 0 .89 | P= 0 .13 | P= 0 .18 | P= 0 .89 |
| KO | 67.516 ± 5.220056 |  |  |  |  |  |  |  |
| WT (B) | 155.43 ± 28.9837 |  |  |  |  |  |  |  |
| KO (B) | 98.5799 ± 26.1949 |  |  |  |  |  |  |  |
| Stained C-FOS cells /mm^2 in medial habenular nucleus (mHab) | | | | | | | | |
|  | Mean ± SEM | Statistics | WT(B)  vs KO(B) | KO(B)  vs KO | KO(B)  vs WT | WT(B)  vs KO | WT(B)  vs WT | WT  vs KO |
| WT | 40.09 ± 6.942933 | One- way ANOVA, Tukey’s posthoc,  F_3,8_= 1.845, P= 0.217 | P= 0 .659 | P= 0 .37 | P= 0 .19 | P= 0 .89 | P= 0 .696 | P= 0 .89 |
| KO | 50.196 ± 9.72688 |  |  |  |  |  |  |  |
| WT (B) | 62.3207 ± 2.254 |  |  |  |  |  |  |  |
| KO (B) | 86.0605 ± 20.4639 |  |  |  |  |  |  |  |
| Stained C-FOS cells /mm^2 in medial dorsal thalamus (MD) | | | | | | | | |
|  | Mean ± SEM | Statistics | WT(B)  vs KO(B) | KO(B)  vs KO | KO(B)  vs WT | WT(B)  vs KO | WT(B)  vs WT | WT  vs KO |
| WT | 57.5109 ± 7.35708 | One- way ANOVA, Tukey’s posthoc,  F_3,8_= 3.24, P= 0.081 | P= 0 .27 | P= 0 .77 | P= 0 .89 | P= 0 .08 | P= 0 .15 | P= 0 .89 |
| KO | 36.6868 ± 7.03762 |  |  |  |  |  |  |  |
| WT (B) | 160.7989 ± 44.929 |  |  |  |  |  |  |  |
| KO (B) | 75.2857 ± 17.9898 |  |  |  |  |  |  |  |
| Stained C-FOS cells /mm^2 in periaqueductal gray (PAG) | | | | | | | | |
|  | Mean ± SEM | Statistics | WT(B)  vs KO(B) | KO(B)  vs KO | KO(B)  vs WT | WT(B)  vs KO | WT(B)  vs WT | WT  vs KO |
| WT | 53.1601 ± 9.01798 | One- way ANOVA, Tukey’s posthoc,  F_3,8_= 21.64, P= 0.0003 | P= 0.3 | P= 0 .001 | P= 0 .001 | P= 0 .006 | P= 0 .011 | P= 0 .89 |
| KO | 43.2379 ± 5.32276 |  |  |  |  |  |  |  |
| WT (B) | 134.599 ± 13.1645 |  |  |  |  |  |  |  |
| KO (B) | 170.08 ± 13.7955 |  |  |  |  |  |  |  |
| Stained C-FOS cells /mm^2 in anterior paraventricular Thalamus (aPVT) | | | | | | | | |
|  | Mean ± SEM | Statistics | WT(B)  vs KO(B) | KO(B)  vs KO | KO(B)  vs WT | WT(B)  vs KO | WT(B)  vs WT | WT  vs KO |
| WT | 64.460 ± 11.45138 | One- way ANOVA, Tukey’s posthoc,  F_3,8_= 7.74, P= 0.0095 | P= 0 .75 | P= 0 .17 | P= 0 .046 | P= 0 .043 | P= 0 .013 | P= 0 .79 |
| KO | 109.49 ± 4.920257 |  |  |  |  |  |  |  |
| WT (B) | 274.9 ± 54.27367 |  |  |  |  |  |  |  |
| KO (B) | 227.0164 ± 15.375 |  |  |  |  |  |  |  |
