# Supplementary material for: IQSEC2 Deficiency Results in Abnormal Social Behaviors Relevant to Autism by Affecting Functions of Neural Circuits in the Medial Prefrontal Cortex
Source: Cells. 2021 Oct 12;10(10):2724. doi: 10.3390/cells10102724 (PMC8534507; doi:10.3390/cells10102724)
Supplement: Supplementary file 1 [file cells-10-02724-s001.zip › Table S4.docx]

Table S4: Results of Data Analysis (2).

| Figure | Parameter | Statistics |
| --- | --- | --- |
| 5B | Frequency of mEPSC | One- way ANOVA, Bonferroni posthoc,  F_2,28_= 30.44, P= 9.5E-8 |
|  | Amplitude of mEPSCs | One- way ANOVA, Bonferroni posthoc,  F_2,28_= 1.72, P= 0.197 |
| 5C | Frequency of mIPSC | One- way ANOVA, Bonferroni posthoc,  F_2,29_= 9.32, P= 0.0008 |
|  | Amplitude of mIPSCs | One- way ANOVA, Bonferroni posthoc,  F_2,29_= 1.22, P= 0.31 |
| 5D | excitatory PPR | One- way ANOVA, Bonferroni posthoc,  F_2,26_= 7.99, P= 0.002 |
| 5E | AMPA EPSC Amplitude | One- way ANOVA, Bonferroni posthoc,  F_2,37_= 6.33, P= 0.0043 |
| 5F | GABA IPSC Amplitude | One- way ANOVA, Bonferroni posthoc,  F_2,44_= 4.85, P= 0.0125 |
| 5G | NMDA/AMPA ratio | One- way ANOVA, Bonferroni posthoc,  F_2,28_= 5.72, P= 0.0083 |
| 6A | Interaction time with Juvenile mouse | One- way ANOVA, Tukey’s posthoc,  F_2,33_= 5.4, P= 0.0093 |
| 6B | Sociability  (S1-E) | One- way ANOVA, Tukey’s posthoc,  F_2,34_= 11.44, P= 0.0002 |
| 6C | Social Novelty  (S2-S1) | One- way ANOVA, Tukey’s posthoc,  F_2,34_= 9.07, P= 0.0007 |
