# Supplementary figures and images for: IQSEC2 Deficiency Results in Abnormal Social Behaviors Relevant to Autism by Affecting Functions of Neural Circuits in the Medial Prefrontal Cortex
Source: Cells. 2021 Oct 12;10(10):2724. doi: 10.3390/cells10102724 (PMC8534507; doi:10.3390/cells10102724)

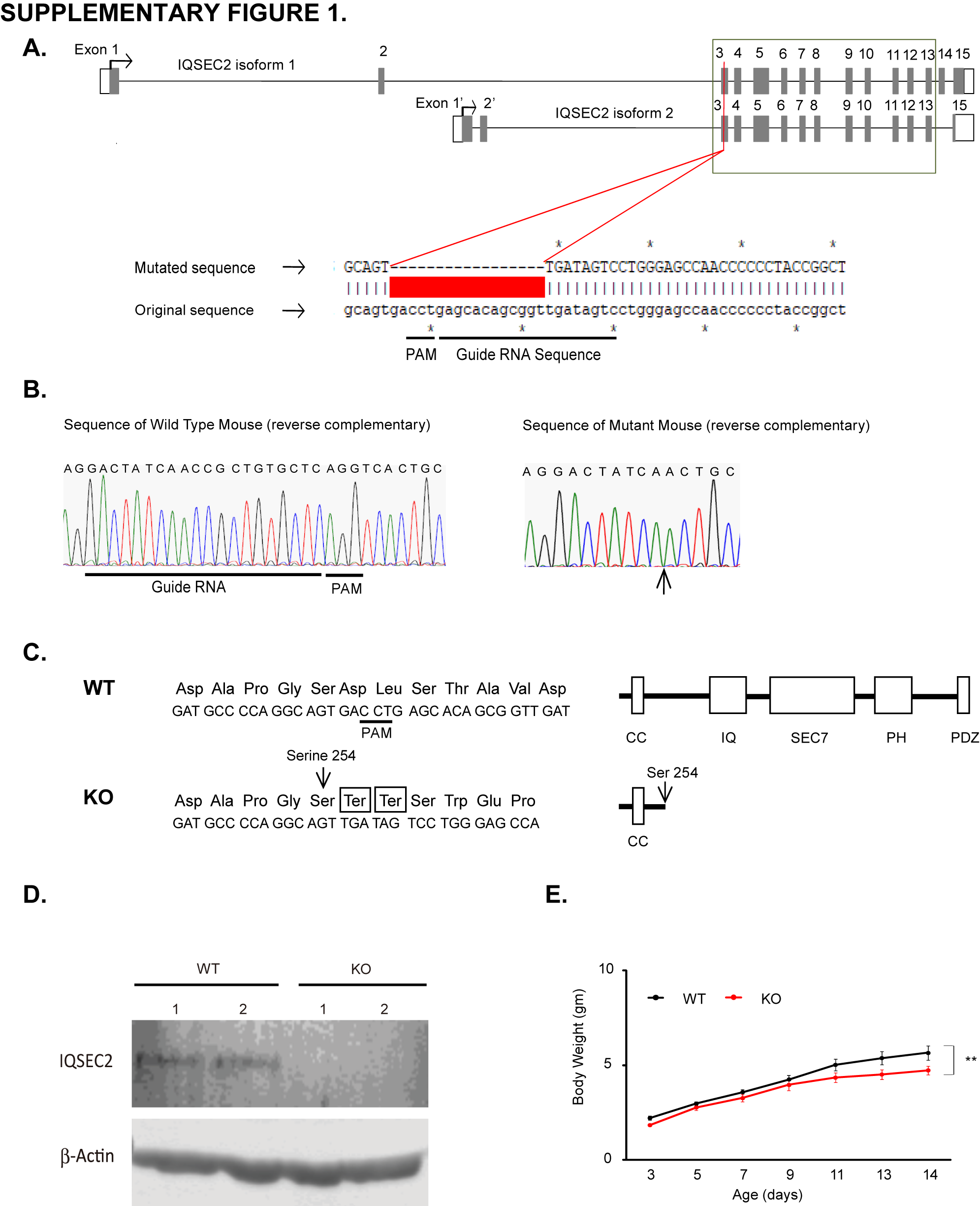

Supplement: Supplementary file 1 [file cells-10-02724-s001.zip › Fig S1.tif]

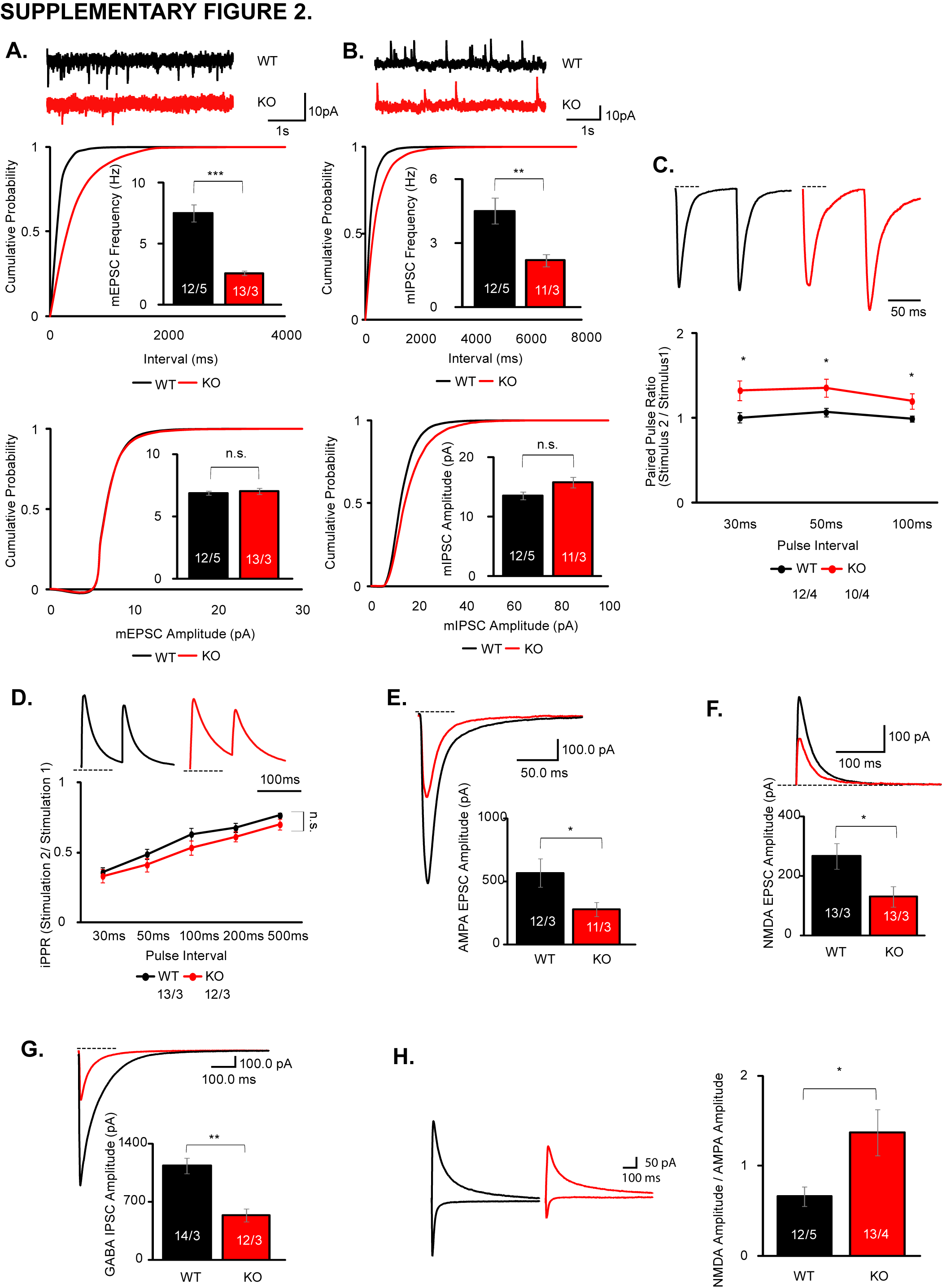

Supplement: Supplementary file 1 [file cells-10-02724-s001.zip › Fig S2.tif]

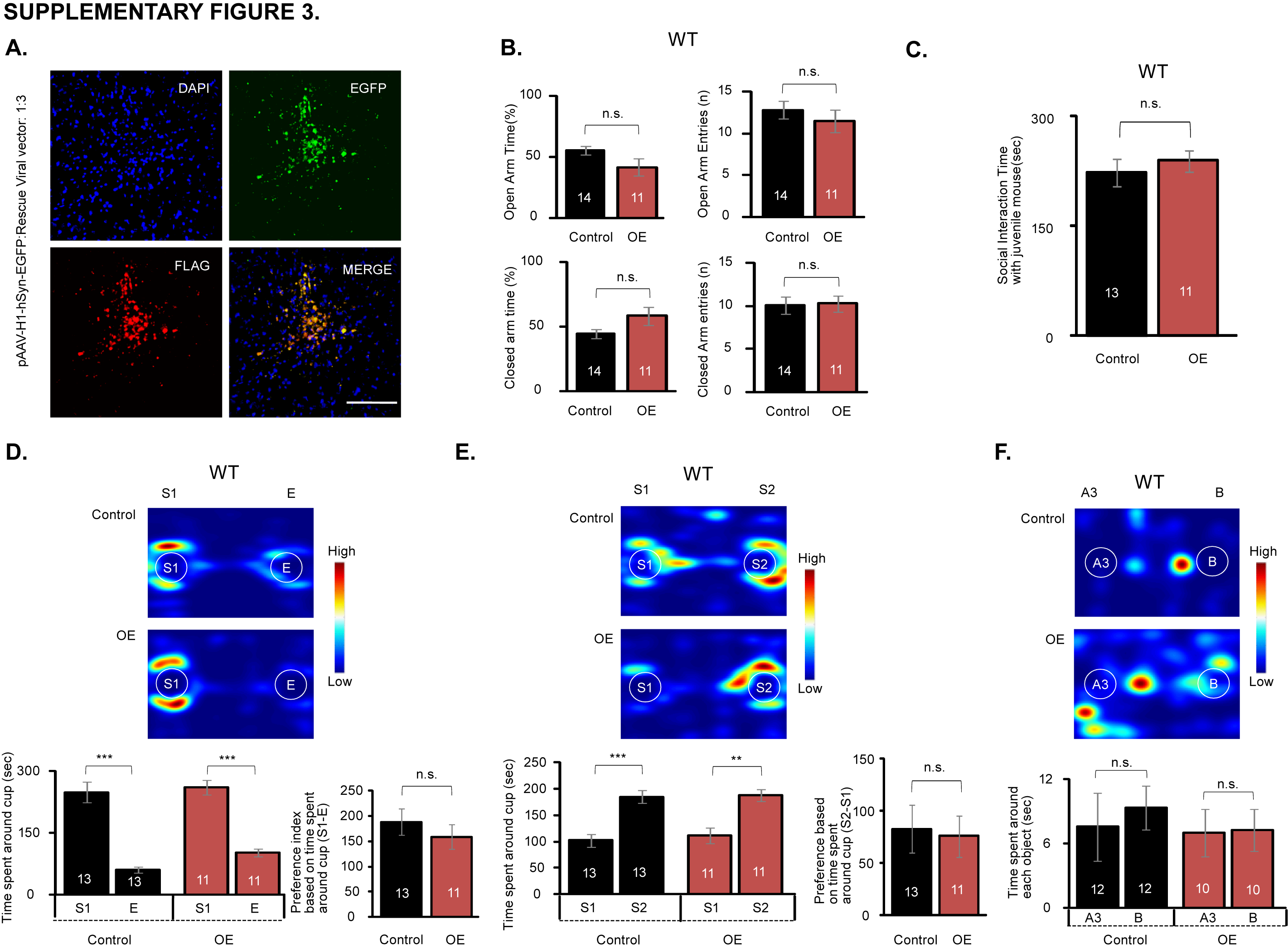

Supplement: Supplementary file 1 [file cells-10-02724-s001.zip › Fig S3.tif]

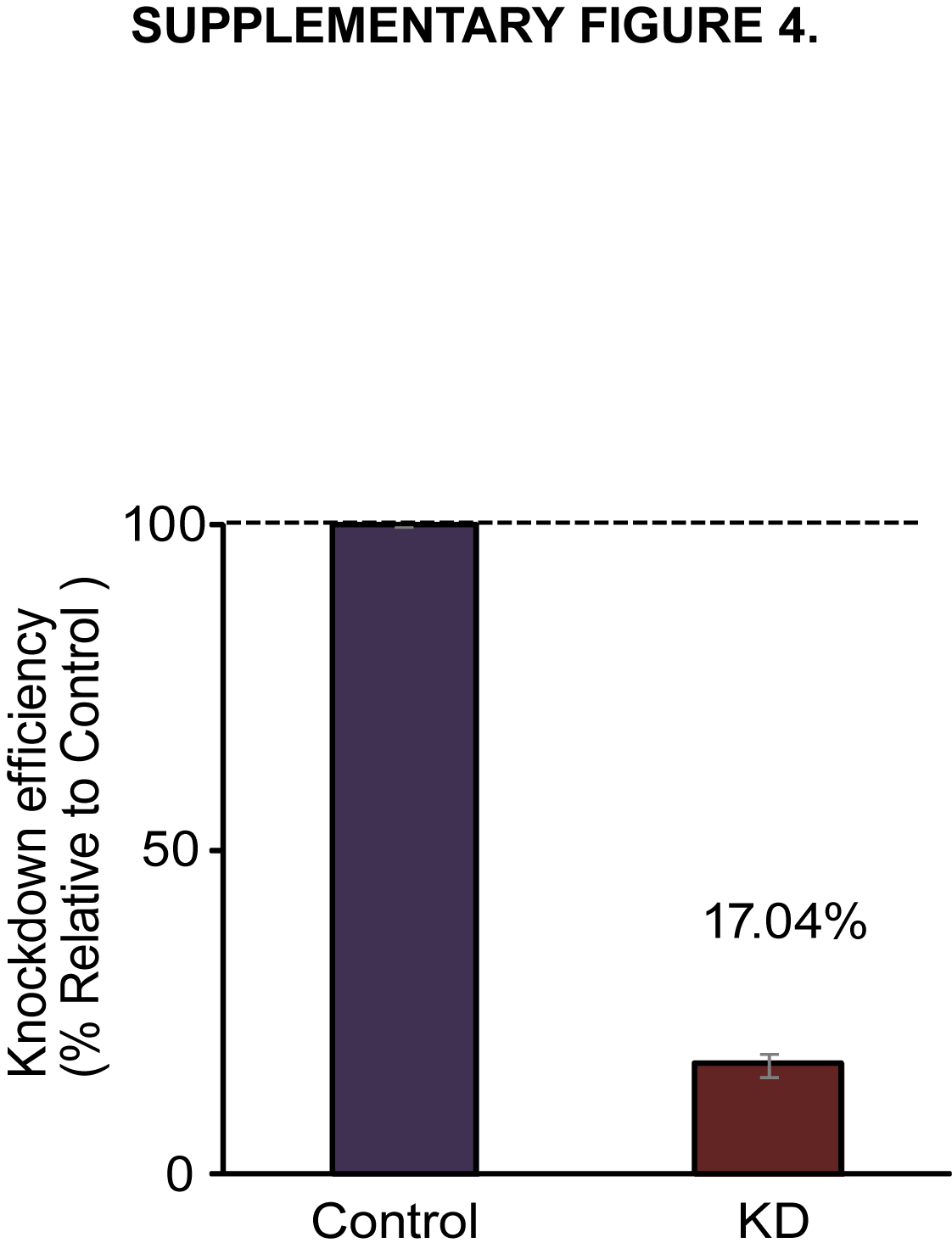

Supplement: Supplementary file 1 [file cells-10-02724-s001.zip › Fig S4.tif]
